# Apremilast for biologic-naïve, peripheral psoriatic arthritis, including patients with early disease: results from the APROACH observational prospective study

**DOI:** 10.1007/s00296-022-05269-z

**Published:** 2023-03-01

**Authors:** Petros P. Sfikakis, Dimitrios Vassilopoulos, Gkikas Katsifis, Georgios Vosvotekas, Theodoros Dimitroulas, Prodromos Sidiropoulos, Periklis Vounotrypidis, Dimitrios P. Bogdanos, Athanasios Ι. Georgountzos, Andreas G. Bounas, Panagiotis Georgiou, Souzana Gazi, Evangelia Kataxaki, Stamatis-Nick Liossis, Evangelos Theodorou, Charalampos Papagoras, Evangelos Theotikos, Panayiotis Vlachoyiannopoulos, Paraskevi V. Voulgari, Angeliki Kekki, Nikolaos Antonakopoulos, Dimitrios T. Boumpas

**Affiliations:** 1 Department of Propedeutic Internal Medicine, Medical School, National and Kapodistrian University of Athens, 17 Agiou Thoma Str., 115 27 Athens, Greece; 2 Medical School, Joint Academic Rheumatology Program, National and Kapodistrian University of Athens, 75 Mikras Asias Street, 115 27 Athens, Greece; 3 Department of Medicine and Clinical Immunology-Rheumatology Unit, Medical School, National and Kapodistrian University of Athens, 114 Vass. Sophias Ave., 115 27 Athens, Greece; 4 Naval Hospital of Athens, 70 Dinokratous Str., 115 21 Athens, Greece; 5 Euromedica General Clinic of Thessaloniki, 11 Maria Kallas Str., 546 45 Thessaloniki, Greece; 6 Department of Internal Medicine, Medical School, Hippokration Hospital, Aristotle University of Thessaloniki, 49 Konstantinoupoleos Str., 546 42 Thessaloniki, Greece; 7 Rheumatology, Clinical Immunology and Allergy, Medical School, University of Crete, Voutes, Crete, 711 10 Heraklion, Greece; 8 Department of Rheumatology, 424 General Army Hospital, Nea Efkarpia, 564 29 Thessaloniki, Greece; 9 Department of Internal Medicine, Faculty of Medicine, School of Health Sciences, University Hospital of Larissa, University of Thessaly, Mezourlo, 411 10 Larissa, Greece; 10 General Hospital of Athens G. Gennimatas, 154 Mesogeion Ave., 115 27 Athens, Greece; 11 Olympion Private General Clinic of Patras, Volou & Meilichou Str., 264 43 Patras, Greece; 12 Rheumatology Unit, Agios Andreas Hospital, 37 Kalavriton Str., 263 32 Patras, Greece; 13 Department of Rheumatology, KAT General Hospital of Attica, 2 Nikis Str., Kifissia, 145 61 Athens, Greece; 14 Rheumatology Unit, Thriasio General Hospital of Elefsina, G. Gennimata Ave., 196 00 Magoula, Greece; 15 Division of Rheumatology, Department of Internal Medicine, Medical School, Patras University Hospital, University of Patras, Rio Achaia, 265 04 Patras, Greece; 16 Rheumatology Clinic 251 Hellenic Air Force Hospital, 3 Panagioti Kanellopoulou Ave., 115 25 Athens, Greece; 17 First Department of Internal Medicine, Medical School, University Hospital of Alexandroupolis, Democritus University of Thrace, 681 00 Alexandroupolis, Greece; 18 Rheumatology Department, Asklepieion Voulas General Hospital, 1 Vasileos Pavlou Ave, 166 73 Athens, Greece; 19 Department of Pathophysiology, Medical School, National and Kapodistrian University of Athens, 17 Agiou Thoma Str., 115 27 Athens, Greece; 20 Department of Rheumatology, School of Health Sciences, Faculty of Medicine, University of Ioannina, 451 10 Ioannina, Greece; 21 Genesis Pharma SA, Athens, 274 Kifissias Ave., 152 32 Halandri, Greece; 22 Department of Internal Medicine, Medical School, “Attikon” University Hospital, Athens, National and Kapodistrian University of Athens, 1 Rimini Str., 124 62 Athens, Greece

**Keywords:** Psoriatic arthritis, Apremilast, Disease activity, Enthesitis, Dactylitis, PsAID12

## Abstract

To evaluate the effect of the phosphodiesterase 4 inhibitor apremilast in biologic-naïve patients with early peripheral PsA in terms of disease activity, clinical manifestations, patient-perceived outcomes, as well as apremilast’s safety profile in routine care settings of Greece. Non-interventional, multicenter, 52-week prospective cohort study, enrolling biologic-naïve patients with early active peripheral PsA who started apremilast after intolerance or inadequate response (within the first 12 months of treatment) to an initial conventional synthetic (cs)DMARD treatment. Non-responder imputation was applied for missing data.In total, 167 consecutive patients (mean age: 52.5 years; median PsA duration: 0.9 years) were analyzed. At baseline, the median (interquartile range) clinical Disease Activity in Psoriatic Arthritis (cDAPSA) score was 22.0 (16.0–29.0), with 86.8% of patients having at least moderate (29.3% high) disease activity; 87.4% had skin psoriasis, 37.7% nail psoriasis, 30.7% enthesitis, and 12.4% dactylitis. At 16, 24, and 52 weeks, 28.7, 42.5, and 48.5% of patients, achieved ≥ 50% improvement in their baseline cDAPSA score, respectively. At week 52, 55.6, 50, and 26.8% of evaluable patients achieved complete resolution of enthesitis, dactylitis and nail psoriasis, respectively. Improvements were also observed in patient’s health state assessed by the Psoriatic Arthritis Impact of Disease 12-item questionnaire, and health-related quality of life. The 52-week drug survival rate was 75%, while 13.8% of patients experienced at least one adverse drug reaction.Biologic-naïve patients with early PsA, treated with apremilast experienced significant improvements in disease activity, extra-articular manifestations and patient-centered outcomes, accompanied by a favorable tolerability profile.

**Supplementary Information:**

The online version contains supplementary material available at 10.1007/s00296-022-05269-z.

## Background

Psoriatic arthritis (PsA) is a heterogeneous inflammatory joint disease with complex pathophysiology and a wide spectrum of musculoskeletal and dermatological manifestations, such as peripheral arthritis, axial disease, dactylitis, enthesitis, skin, and nail involvement [1–4]. PsA has been associated with various comorbid conditions and has a negative impact on patients’ emotional state, personal and social relationships, daily activities, and health-related quality of life (HRQoL) [2, 5–8].

The target of PsA therapy is achievement of remission or low disease activity [9], according to composite indices, such as Disease Activity Index for Psoriatic Arthritis (DAPSA) and clinical DAPSA (cDAPSA) used to assess response to treatment. Pharmacological treatment options for PsA include non-steroidal anti-inflammatory drugs (NSAIDs), glucocorticoids, and disease-modifying anti-rheumatic drugs (DMARDs), which are classified into conventional synthetic (csDMARDs), biologic (bDMARDs), and targeted synthetic (tsDMARDs) [9–12]. The latter are generally used if other options fail to achieve treatment target or are considered otherwise inappropriate [9].

The novel phosphodiesterase 4 inhibitor apremilast is the first oral tsDMARD approved for PsA and is indicated for adults with active disease and inadequate response or intolerance to a prior DMARD [13, 14]. Apremilast has demonstrated clinical efficacy accompanied by a favorable safety and tolerability profile in the product’s clinical trial program across a wide spectrum of PsA patient profiles [10, 15–20]. In these trials, efficacy was primarily assessed on the basis of the proportion of patients achieving at least 20% improvement in modified American College of Rheumatology (ACR) response criteria, which was significantly higher in the apremilast- than in the placebo-receiving groups. Other efficacy outcomes included improvements in signs and symptoms of PsA, in physical function, as well as in the severity of PsA manifestations such as enthesitis, dactylitis, skin, and nail psoriasis. The positive effects of apremilast were observed both in biologic-experienced and biologic-naïve patients, while improvements were noted as early as after 16 weeks of treatment [10, 15–20] and sustained for up to 5 years [18].

To complement data derived from clinical trials, the APROACH study aimed to generate real-world evidence on the effect of apremilast across various clinical and patient-reported outcomes (PROs) in a representative sample of biologic-naïve patients with early PsA treated under real-life conditions in Greece.

## Methods

### Study design and population

APROACH was a non-interventional, multicenter, 52-week prospective cohort study. Eligible subjects were adults with physician-diagnosed active peripheral PsA, with inadequate response, as judged by the treating physician (within the first 12 months of treatment) or intolerance to prior cDMARD therapy who were prescribed for the first time apremilast for PsA according to the approved label. cDMARDs were continued or not as per physician’s discretion. Patients already initiated on apremilast could be considered for enrollment provided ≤ 1 week had elapsed from treatment initiation to informed consent. Available information on the cDAPSA components was a prerequisite for study enrollment. Patients previously exposed to biologics or tofacitinib, those having received investigational products within 30 days or 5 half-lives of the investigational agent before the start of apremilast therapy, as well as pregnant and lactating females were excluded.

Primary data were collected by routine clinical assessments, patient report, and from medical records in four visits taking place at enrollment and at 16 ± 3, 24 ± 3, and 52 ± 4 weeks after baseline, defined as the time of apremilast initiation. Physicians were requested to consecutively enroll the first eligible patients attending their clinic, and follow each participant for a 52-week observation period post-baseline or until withdrawal of consent, discontinuation of apremilast, physician’s decision or the patient was no longer considered eligible for participation, whichever occurred earlier.

The study was conducted according to the principles of the international society for pharmacoepidemiology guidelines for good pharmacoepidemiology practice, the ethical principles laid down in the declaration of Helsinki, the Strengthening the Reporting of Observational Studies in Epidemiology guidelines, and all applicable local rules and regulations. The study protocol was approved by the institutional review board of each participating study site. The first protocol approval was obtained on January 15, 2019 by the ethics committee of the Athens Naval Hospital, with ethics approval protocol number 1/19.

### Assessments and definitions

The clinical indices of disease activity and extra-articular manifestations [66-Swollen Joint Count (SJC), 68-Tender Joint Count (TJC), Dactylitis Severity Score (DSS), Leeds Enthesitis Index (LEI), Nail Physician Global Assessment (PhGA), psoriasis-affected Body Surface Area (BSA), PhGA of patient’s general health], and C-reactive protein (CRP) were collected at baseline and throughout study participation, according to study visit schedule, and as performed per routine practice. PROs used for addressing the objectives presented herein included Patient Global Assessment (PtGA) of Rheumatic Disease Activity and PtGA of Joint Pain [both are 11-point numerical rating scales (NRS)], Psoriatic Arthritis Impact of Disease 12-item (PsAID12), EuroQol 5-Dimensions 5-Levels (EQ-5D-5L), and Health Assessment Questionnaire-Disability Index (HAQ-DI), collected via self-completed paper questionnaires. PtGA of Rheumatic Disease Activity, PtGA of Joint Pain, 66-SJC, and 68-TJC are used for the calculation of the cDAPSA composite score (range: 0–154), while, combined with CRP, they are used for the DAPSA score calculation (range: 0–164). Achievement of minimal disease activity was defined as the fulfillment of ≥ 5 of the following 7 measures: TJC ≤ 1, SJC ≤ 1, BSA ≤ 3%; PtGA-Joint Pain NRS ≤ 1, PtGA-Rheumatic Disease Activity NRS ≤ 2.0, HAQ-DI score ≤ 0.5, and LEI ≤ 1.

Minor, moderate, and major cDAPSA response are defined as ≥ 50%, ≥ 75%, and ≥ 85% improvement in baseline cDAPSA score, respectively. Based on cDAPSA, patients were classified as being in remission (score ≤ 4), and as having low (LDA), moderate (MDA), or high disease activity (HDA) when their score was > 4 but ≤ 13, > 13 but ≤ 27, and > 27, respectively [21].

### Study objectives

The primary study objective was to evaluate the impact of apremilast on peripheral PsA disease activity at week 24 by estimating the minor response rate using the cDAPSA composite index. Secondary objectives included the estimation of the 52-week minor cDAPSA response rate, the 24- and 52-week moderate and major cDAPSA response rates, as well as the evaluation of the effect of apremilast treatment on enthesitis, dactylitis, skin and nail psoriasis at weeks 24 and 52 among patients affected at baseline. Additional secondary objectives presented herein were to assess the effect of apremilast treatment on the impact of PsA on several aspects of patients’ daily living and generic HRQoL at weeks 16 and 52, as assessed by the European League Against Rheumatism PsAID12 and the EQ-5D-5L questionnaires, respectively, and to evaluate the 52-week drug survival rate and the safety profile of apremilast in a real-world setting. Furthermore, predictors of attainment of minor cDAPSA response at 52 weeks post-baseline were examined.

### Statistical considerations

Sample size was calculated based on the primary objective. Assuming an approximate 30% non-evaluable/drop-out rate, 170 enrolled patients (119 evaluable) were considered sufficient to estimate a 24-week minor cDAPSA response rate of 50% with a margin of error [half-width of confidence interval (CI)] not exceeding 9%.

The primary endpoint and secondary endpoints addressing response rates were analyzed using a modified non-responder imputation (NRI), in which patients with missing data for any reason other than disease remission were considered as non-responders. Data as-observed are also provided. For binomial proportions (including the primary endpoint analysis), 95% CI were derived from Wald confidence limits. The normality of distribution of continuous variables was examined using the Shapiro–Wilk test. Statistical significance of changes from baseline was examined using paired t-test, Wilcoxon signed-rank test, or McNemar’s test, as applicable. Drug survival was analyzed using the Kaplan–Meier method.

For the analysis of the association of factors of interest with achievement of ≥ 50% improvement in cDAPSA baseline score at week 52, NRI data were used and the multivariable logistic regression model presented was derived from a stepwise procedure based on the minimization of Akaike’s information criterion. The initial step included age, sex, place of residence, obesity, presence of comorbidities, duration of PsA, number of affected joints, presence of nail psoriasis, presence of dactylitis and/or enthesitis at baseline, apremilast initiated as monotherapy or combination with DMARD, week-16 minor cDAPSA response, and oligo-plus disease defined as monoarthritis or oligoarthritis plus any extraarticular manifestation (enthesitis/dactylitis/skin psoriasis/nail psoriasis).

All statistical tests were two-sided and performed at a 0.05 significance level. The sample size determination and statistical analyses were performed using SAS^®^ (v.9.4; SAS Institute, Cary, NC).

## Results

### Patient disposition

**Fig. 1:**
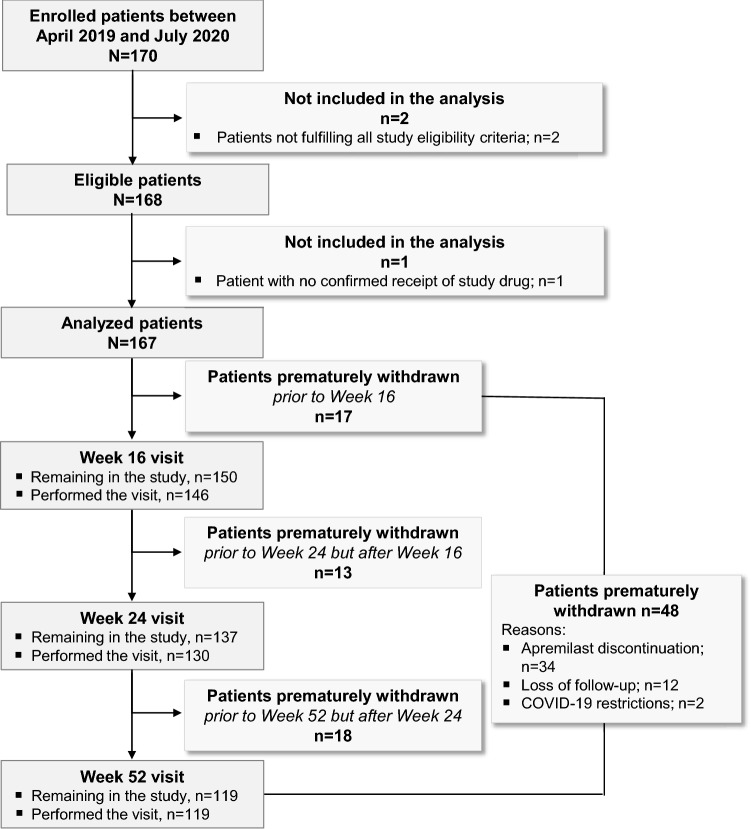
Patient disposition

Between 15-Apr-2019 and 06-Jul-2020, 170 patients were enrolled by rheumatology specialists in 20 public or private hospital centers/clinics, of whom 167 were analyzed (Fig. 1). The median (interquartile range, IQR) duration of study participation for the overall analyzed population was 52.0 (35.9–52.9) weeks.

### Baseline characteristics and prior treatments

Patient baseline characteristics are shown in Table 1. The mean (standard deviation, SD) age at skin manifestation onset was 40.4 (16.1) years. Before apremilast initiation, all patients had received csDMARDs; 59.3% had also received other therapies, comprising oral NSAIDs (34.7%), topical treatments (16.8%), systemic steroids (14.4%), folic acid (7.8%), intra-articular steroid injections (1.8%), and photo(chemo)therapy, acitretin, and pregabalin (0.6% each). The most common prior csDMARD in the study population was methotrexate, received by 93.4% and discontinued in 52.1% prior to baseline. The main reasons for discontinuation of methotrexate were adverse events (AE)/intolerance (46/87 who discontinued) or inadequate/loss of response (33/87). At baseline, 53.3% (89/167) of the patients had disease duration < 1 year.

**Table 1:** Baseline characteristics of the overall eligible population and the subpopulation attending the Week 52 visit

	Overall eligible population (*N* = 167)		Patients who completed 52 weeks of observation (*N* = 119)	
	*N*		*N*	
Baseline characteristics				
Caucasian, *n* (%)	167	167 (100.0)	119	119 (100.0)
Females, *n* (%)	167	103 (61.7)	119	74 (62.2)
Age at baseline, years, mean (SD)	167	52.5 (12.3)	119	54.5 (12.4)
Place of residence: urban area, *n* (%)	158	131 (82.9)	114	94 (82.5)
Education ≤ 12 years, *n* (%)	137	75 (54.7)	95	53 (55.8)
Full or part-time/self- employed, n (%)	143	92 (64.3)	100	60 (60.0)
BMI, kg/m^2^, median (IQR)	142	27.2 (24.6–31.2)	100	26.9 (24.7–30.3)
Obese (BMI ≥ 30 kg/m^2^), *n* (%)	142	46 (32.4)	100	29 (29.0)
Clinically significant medical/surgical history or comorbidities, *n* (%)	167	74 (44.3)	119	43 (36.1)
Comorbidities in ≥ 10% of overall population				
Hypertension, *n* (%)	167	32 (19.2)	119	24 (20.2)
Dyslipidemia, *n* (%)	167	18 (10.8)	119	9 (7.6)
Disease characteristics				
Age at PsA diagnosis, years, mean (SD)	167	50.8 (12.3)	119	52.6 (12.4)
PsA duration at baseline, years, median (IQR)	167	0.9 (0.5–1.7)	119	1.1 (0.7–1.8)
PsA clinical type at baseline				
Peripheral joint involvement only, *n* (%)	167	155 (92.8)	119	110 (92.4)
Predominant peripheral joint with coexisting axial involvement, *n* (%)	167	9 (5.4)	119	7 (5.9)
Predominant axial with coexisting peripheral joint involvement, *n* (%)	167	3 (1.8)	119	2 (1.7)
PsA clinical subtype at baseline				
Polyarthritis (≥ 5 joints), *n* (%)	167	113 (67.7)	119	78 (65.5)
Oligoarthritis (2–4 joints), *n* (%)	167	45 (26.9)	119	35 (29.4)
Monoarthritis (1 joint), *n* (%)	167	7 (4.2)	119	4 (3.4)
Distal interphalangeal joint arthritis, *n* (%)	167	3 (1.8)	119	2 (1.7)
Predominant spondylitis, *n* (%)	167	3 (1.8)	119	2 (1.7)
DAPSA score, median (IQR)	132	24.4 (18.8–31.8)	92	24.7 (20.1–32.0)
cDAPSA score, median (IQR)	167	22.0 (16.0–29.0)	119	23.0 (17.0–30.0)
Number of swollen joints, median (IQR)	167	4.0 (2.0–8.0)	119	4.0 (2.0–8.0)
Number of tender joints, median (IQR)	167	5.0 (2.0–9.0)	119	6.0 (2.0–9.0)
CRP levels, mg/dL, median (IQR)	132	1.0 (0.5–3.0)	92	1.0 (0.6–2.7)
Active skin psoriasis (BSA > 0%), *n* (%)	167	146 (87.4)	119	108 (90.8)
Nail involvement, *n* (%)	162	61 (37.7)	116	51 (44.0)
Enthesitis, *n* (%)	163	50 (30.7)	115	34 (29.6)
Dactylitis, *n* (%)	161	20 (12.4)	115	12 (10.4)
Active skin psoriasis and/or nail involvement, and/or dactylitis, and/or enthesitis, *n* (%)	166	158 (95.2)	118	114 (96.6)
Treatment characteristics				
Prior csDMARD therapy for PsA, *n* (%)	167	167 (100.0)	119	119 (100.0)
Methotrexate, *n* (%)	167	156 (93.4)	119	110 (92.4)
Leflunomide, (%)	167	18 (10.8)	119	15 (12.6)
Ciclosporin, (%)	167	16 (9.6)	119	11 (9.2)
Apremilast initiated combined with csDMARD, *n* (%)	167	87 (52.1)	119	60 (50.4)
Methotrexate, (%)	167	76 (45.5)	119	50 (42.0)
Leflunomide, (%)	167	11 (6.6)	119	10 (8.4)

*BMI* body mass index, *BSA* body surface area, *cDAPSA* *clinical disease activity in psoriatic arthritis*, *CRP* C-reactive protein, *csDMARD* conventional synthetic disease-modifying antirheumatic drug, *IQR* interquartile range, *N* number of patients with available data, *PsA* psoriatic arthritis, *SD* standard deviation

### Apremilast treatment

Apremilast was initiated according to the recommended 5-day morning and evening titration schedule, followed by a 30 mg twice daily maintenance dose schedule in all patients. The time elapsed from apremilast initiation to enrollment ranged from –7 to 17 days, with treatment having started before enrollment in 49 patients (29.3%), on the date of enrollment in 64 patients (38.3%), and after enrollment in 54 patients (32.3%). In 46.7% it was initiated as monotherapy and in 53.3% combined with other pharmacological therapies (methotrexate in 76 patients, leflunomide in 11, and prednisolone and etoricoxib in one each).

During the study observation period, the median (IQR) length of exposure to apremilast was 12.0 (8.2–12.2) months; 30.4% (48/158) of patients with available data permanently discontinued apremilast treatment; for 13 of these patients, the decision for apremilast discontinuation was made on the day of their 52-week visit. The reasons for apremilast discontinuation were lack of efficacy for 24 patients, patient’s decision for 14 patients, and AE for the remaining ten patients (see Additional file 1). The Kaplan–Meier-estimated 52-week apremilast continuation rate was 75% (95% CI: 67.4–81.1) (see Additional file 2).

### Effect of apremilast on psoriatic arthritis disease activity

**Fig. 2:**
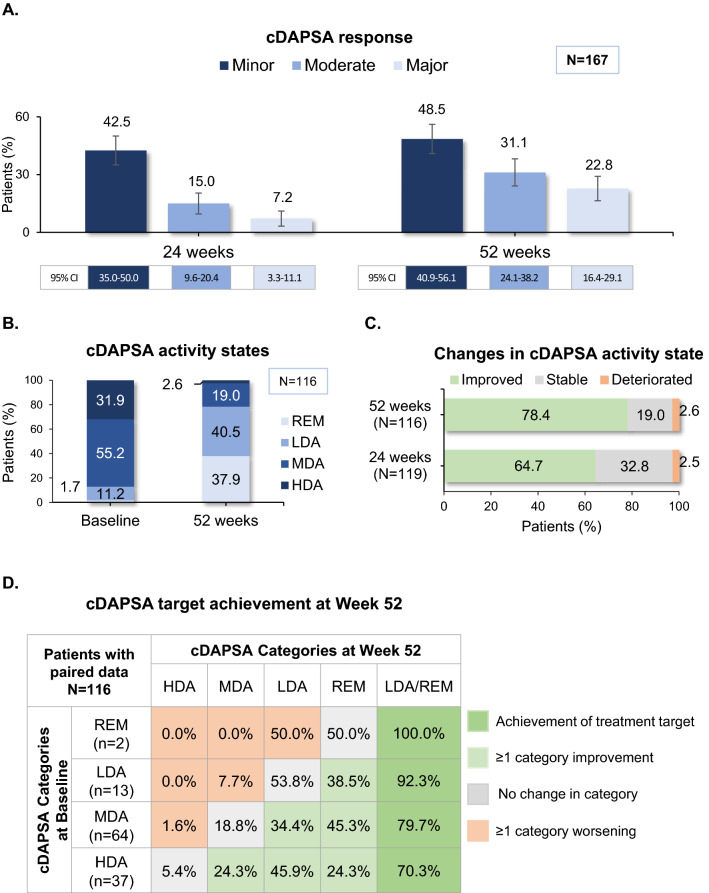
Effect of apremilast on disease activity. **A** Minor, moderate, and major cDAPSA response at 24 and 52 weeks. Error bars indicate 95% confidence intervals. **B** cDAPSA activity states at baseline and 52 weeks post-baseline in patients with paired data. **C** Changes in cDAPSA activity states at 24 and 52 weeks post-baseline in patients with paired data. **D** Achievement of cDAPSA target at 52 weeks post-baseline in patients with paired data. *cDAPSA* clinical disease activity in psoriatic arthritis, *CI* confidence interval, *HDA* high disease activity, *MDA* moderate disease activity, *LDA* low disease activity, *N* number of patients with available data, *n* number of patients in category, *REM* remission

Based on as-observed data, minor cDAPSA response rate was 59.7% (71/119) at 24, and 69.8% (81/116) at 52 weeks post-baseline, with 42.5% (48/113) of patients (28.7% NRI rate) having attained such response already at 16 weeks. Moderate and major cDAPSA response rate was 21.0% (25/119) and 10.1% (12/119) at 24 weeks, and 44.8% (52/116), and 32.8% (38/116) at 52 weeks, respectively. NRI-analyzed minor cDAPSA response rate was 42.5% at 24, and 48.5% at 52 weeks post-baseline; the respective moderate and major response rates were 15.0 and 7.2% at 24, and 31.1% and 22.8% at 52 weeks post-baseline (Fig. 2A).

According to cDAPSA, the proportion of patients classified as having at least moderate disease activity was 86.8% (145/167) at baseline (including 49 patients with HDA), 35.3% (42/119) at 24 weeks (including seven with HDA), and 21.6% (25/116) at 52 weeks post-baseline (including three with HDA). Changes in cDAPSA activity state from baseline at 24 and 52 weeks among patients with available paired data are depicted in Fig. 2B–D.

Based on the 66-SJC, patients had a median number of 4.0 (2.0–8.0), 2.0 (1.0–4.0), and 0.0 (0.0–2.0) swollen joints, at baseline, 16, and 52 weeks post-baseline, respectively. Among patients with available paired data and SJC > 0 at baseline (*N* = 140 at 16 weeks, and *N* = 113 at 52 weeks), a statistically significant median (IQR) decrease of 50.0% (0.0–75.0%) and 90.0% (60.0–100%) in the SJC was observed at 16, and 52 weeks post-baseline, respectively (*p* < 0.001).

Based on the 68-TJC, patients had a median number of 5.0 (2.0–9.0), 2.0 (1.0–5.0), and 1.5 (0.0–3.0) tender joints, at baseline,16, and 52 weeks post-baseline, respectively. Among patients with available paired data TJC > 0 at baseline (*N* = 141 at 16 weeks, and *N* = 112 at 52 weeks), a statistically significant median (IQR) decrease of 50.0% (0.0–75.0%), and 80.0% (50.0–100.0%) in the TJC was observed at 16 and 52 weeks post-baseline, respectively (*p* < 0.001).

Based on as-observed data, 36.9% (45/122) of evaluable patients achieved minimal disease activity at 24 weeks and 55.2% (64/116) at 52 weeks, with the respective NRI-derived rates being 26.9% (45/167) and 38.3% (64/167).

### Effect of apremilast on other psoriatic disease manifestations

At baseline, 87.4% of patients had skin psoriasis (i.e., BSA > 0%), 66.0% (103/156) of patients with available data had BSA > 3%, while 37.7% (61/162) had nail involvement, 30.7% (50/163) had enthesitis (LEI > 0), and 12.4% (20/161) had dactylitis (DSS > 0).

**Fig. 3:**
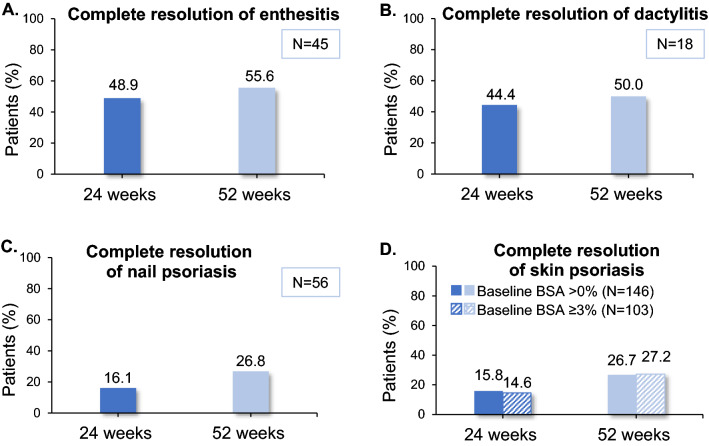
Effect of apremilast on extra-articular manifestations. Proportion of patients with resolution of **A** enthesitis, **B** dactylitis, **C** nail psoriasis, **D** skin psoriasis using NRI imputation at 24 and 52 weeks post-baseline. *BSA* body surface area, *CI* confidence interval, *N* number of patients with available data

Among patients with baseline BSA ≥ 3% and available paired assessments, the median (IQR) BSA score changed from 10.0% (5.0–17.5) at baseline to 3.0% (1.0–5.0) at 24 (*N* = 84) and from 10.0% (5.0–17.0) at baseline to 2.0% (0.0–5.0) at 52 (*N* = 81) weeks (for both comparisons *p* < 0.001). Based on as-observed data, of evaluable patients affected at baseline, 21.4% (9/42) and 34.1% (15/44) achieved complete resolution of nail psoriasis, 64.7% (22/34) and 83.3% (25/30) of enthesitis, and 72.7% (8/11) and 90.0% (9/10) of dactylitis at 24 and 52 weeks, respectively. The respective NRI-derived proportions are presented in Fig. 3.

### Effect of apremilast on the impact of PsA on patients’ daily living

**Fig. 4:**
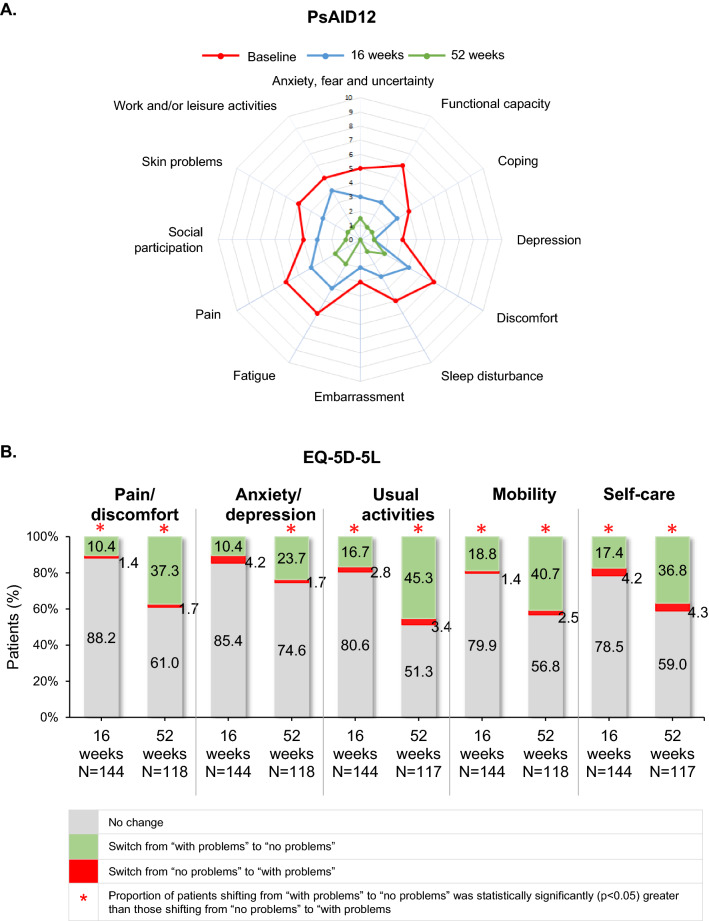
Effect of apremilast on the impact of PsA on patient’s daily living and HRQoL. A. Mean PSAID12 score per domain at 16 and 52 weeks post-baseline. B. Proportions of patients shifting between problems/no problems in EQ-5D-5L from baseline at 16 and 52 weeks post- baseline. *EQ-5D-5L* euroqol 5-dimensions 5-levels, *HRQoL* health-related quality of life, *N* number of patients with available data, *PsA* psoriatic arthritis, *PsAID12* psoriatic arthritis impact of disease 12-item

The median (IQR) total PsAID12 score in the study population at baseline was 5.0 (3.4–6.4) and decreased to 3.6 (2.1–4.5) at 16, and 1.6 (0.4–3.4) at 52 weeks; median item scores are displayed in Fig. 4A. Statistically significant (*p* < 0.001) decreases from baseline were noted both at 16 [mean (SD) decrease: 1.5 (1.6)] and at 52 [median (IQR) decrease: 2.8 (1.1–4.8)] weeks post-baseline; 18.9% (27/143) and 47.4% (55/116) of the patients achieved at least a 3-point reduction in baseline PsAID12 total score at 16 and 52 weeks, respectively.

Improvements were also observed in patients’ generic HRQoL, as assessed by EQ-5D-5L at 16 and 52 weeks post-baseline (Fig. 4B).

### Association of factors of interest with the achievement of minor cDAPSA response at 52 weeks post-baseline

By multivariable analysis, higher age at baseline and achievement of minor cDAPSA response at 16 weeks post-baseline were significantly associated with higher, while obesity and presence of comorbidities at baseline were associated with lower odds of minor cDAPSA response at 52 weeks (see Additional file 3). Initiation of apremilast as monotherapy versus combination with DMARDs was not statistically significantly associated with minor cDAPSA response at 52 weeks (data not shown).

### Safety analysis

The incidence of AEs that occurred from informed consent up to at least 28 days after the last apremilast dose in the context of the study are listed in Table 2. Among the apremilast-related adverse reactions experienced by 13.8% (*n* = 23) of the patients, all but one were considered non-serious, being mainly gastrointestinal complaints (diarrhoea, nausea, etc.) and headache (Table 2). There was only one case of major depression that improved after drug discontinuation.

**Table 2:** Incidence of safety events

Incidence of safety events (*N* = 167)	*n*_event_	*n*_pt_ (%)
Overall safety events (including adverse events and special situations)	124	62 (37.1)
Non-serious	117	60 (35.9)
Serious	7	5 (3.0)
Safety events not causally related to apremilast	45	28 (16.8)
Non-serious	39	26 (15.6)
Serious	6	4 (2.4)
Safety events with unknown causal relation to apremilast	16	11 (6.6)
Non-serious	16	11 (6.6)
Safety events causally related to apremilast	63	36 (21.6)
Non-serious	62	35 (21.0)
Serious	1	1 (0.6)
Apremilast-related adverse events (excluding special situations)	43	23 (13.8)
Non-serious	42	22 (13.2)
Serious	1	1 (0.6)
Description of apremilast-related adverse events by MedDRA v.23.1 preferred term		
Diarrhoea	8	8 (4.8)
Headache	7	7 (4.2)
Nausea	5	5 (3.0)
Fatigue	3	3 (1.8)
Insomnia	3	3 (1.8)
Gastrointestinal disorder	2	2 (1.2)
Abdominal pain	1	1 (0.6)
Anxiety	1	1 (0.6)
Arthritis	1	1 (0.6)
Back pain	1	1 (0.6)
Decreased appetite	1	1 (0.6)
Depression	1	1 (0.6)
Emotional disorder	1	1 (0.6)
Frequent bowel movements	1	1 (0.6)
Gastritis	1	1 (0.6)
Joint effusion	1	1 (0.6)
Major depression^a^	1	1 (0.6)
Persistent depressive disorder	1	1 (0.6)
Photosensitivity reaction	1	1 (0.6)
Vertigo	1	1 (0.6)
Vomiting	1	1 (0.6)
Special situations by MedDRA v.23.1 preferred term		
Drug ineffective	19	19 (11.4)
Condition aggravated	5	5 (3.0)
Intentional product misuse	2	2 (1.2)
Off-label use	2	2 (1.2)

^a^This was the only serious event in the list

*MedDRA* medical dictionary for regulatory activities, *N* total number of patients, *n*_*even*t_ number of events, *n*_*pt*_ number of patients with event

## Discussion

The 52-week real-world study APROACH sheds insight into the effectiveness and safety of apremilast in biologic-naïve patients with early PsA treated in routine care settings in Greece. The characteristics of patients included in APROACH reflect the profile of patient population to whom clinicians had already decided (based on their medical judgment) to prescribe apremilast. Substantial improvement in PsA activity was demonstrated (assessed by cDPASA) as well as in different aspects of psoriatic disease, namely enthesitis, dactylitis, skin, and nail psoriasis. Furthermore, significant reductions in the patient-perceived symptom, physical, and psychosocial disease-related burden, as well as improvements in generic HRQoL were observed.

The study provides evidence on the patient profile and disease characteristics of early peripheral PsA. To date, information on early PsA is limited, with a paucity of published relevant studies. This may contribute to the diagnostic delay and undertreatment of PsA, as well as its substantial burden [7]. Understanding of early PsA is of utmost importance, as it is a phase when treatment may have a more favorable impact on disease progression [22–24]. The question becomes more relevant with the increasing availability of therapeutic options, as identification of predictors of response to treatment or prognostic factors would also be of great value. Thus, evidence generated in APROACH fills a critical gap and at the same time prompts further research in the field.

When viewing the results of APROACH in the context of other apremilast studies, differences in the design and analytical methods should be considered. It should also be highlighted that this study included only biologic-naïve patients, in whom the effect of apremilast may be more pronounced than in biologic-experienced patients [10], who were also included in the pivotal RCTs PALACE 1–3 and real-world observational studies of apremilast described below. The eligibility requirement for patients to be biologic-naïve in APROACH resulted in a short median PsA disease duration of 0.9 years at baseline contrary to other studies including both bio-naïve and bio-experienced patients where the median/mean disease duration at enrollment range was 6.8–35.9 years [10, 15–18, 25–29]. This presumably reflects the current real-world PsA management paradigm in Greece, where patients who are inadequate responders or intolerant to csDMARDs are started earlier treatment with apremilast, without necessitating a steroid bridging therapy.

The primary outcome in APROACH was based on cDAPSA, which, along with DAPSA, has been proposed as valid tools to measure disease activity, response to treatment, and achievement of treatment targets in PsA [30, 31]. The 50% cut-off in (c)DAPSA score improvement (i.e., minor response) is considered to give the best agreement with the ACR20 response [21]. Based on as-observed data, 42.5% of the APROACH population achieved minor cDAPSA response at week 16 and 69.8% (48.5% by NRI) at week 52. These values fall near the upper end of the 32.1–41% 16-week and the 52.6–67.1% 52-week ACR20 range reported in the clinical trial settting, including PALACE 1–3 [15–17], a pooled analysis of PALACE 1–3 [18], the PALACE 4 RCT [19], and the ACTIVE Phase IIIB trial [20]. Improvements in disease activity with apremilast have also been observed in the real-world setting [26, 27, 29].

As cDAPSA mainly focuses on articular symptoms, additional instruments were employed to examine the effect of apremilast on other psoriatic disease manifestations. Overall, 55.6% (assessed by LEI) of patients with enthesitis and 50% of those with dactylitis (assessed by DSS) achieved resolution of these manifestations at week 52. In a pooled analysis of PALACE 1–3 trials [32] 41.1% achieved resolution of enthesitis at week 52, as assessed by the Maastricht Ankylosing Spondylitis Enthesitis Score, whereas among those with dactylitis, 67.5% achieved a dactylitis count of 0 at week 52. In the ACTIVE study, 69.8% of patients with enthesopathy at baseline achieved a Gladman Enthesitis Index score of 0 at 52 weeks [20].

In the real-world setting, based on as-observed data, in the Belgian, multicenter, prospective, study APOLO, among PsA patients with enthesitis and dactylitis at baseline, 37.5% (using LEI), and 71.4% (using dactylitis count) reached a score of 0 at 6 months [26]. Moreover, in APROACH, the median total PsAID12 score decreased from 5.0 at baseline to 1.6 at week 52. The PsAID2 instrument was also used in APOLO, where a decrease from 6.3 at baseline to 4.4 at month 6 was reported among patients with baseline score > 4 [26].

Multivariable analysis in APROACH showed that achievement of minor cDAPSA response at 16 weeks post-baseline was significantly associated with higher odds of minor cDAPSA response at 52 weeks. This is consistent with previous findings from the pooled PALACE 1–3 analysis, which indicated that patients achieving early and at least partial responses, i.e., ≥ 30% improvement in cDAPSA by week 16, had higher probability of achieving treatment targets by week 52 [33].

In APROACH, the minor cDAPSA response rate had a small numerical increase from 24 at 52 weeks (from 42.5 to 48.5%), while the moderate response was > double (from 15.0 to 31.1%) and the major cDAPSA response rate was > three times higher (from 7.2 to 22.8%) at 52 than at 24 weeks. These results may suggest that patients who benefit from apremilast treatment are more likely to display early signs of improvement in disease activity and that it is not so much the proportion of responders that increases, but rather those who respond further deepen their response with continued treatment. In clinical practice, this could minimize the time spent on a treatment that is not suitable for a patient, while the importance of regular disease activity assessments also becomes apparent.

The main limitations of the study are attributed to its observational design and the lack of a comparator arm. A high missing rate for certain patient characteristics and for serum CRP levels limits the evaluable patient populations for specific outcomes. The use of NRI in the response-related outcome analysis may have led to underestimation of apremilast effectiveness, as all patients with missing data are considered non-responders. However, this imputation approach is often chosen, as it is considered more conservative than others. In APROACH, no retrospective AE collection took place for patients having initiated apremilast before informed consent; thus, the incidence of AEs may have been underestimated, particularly those more likely to occur during the first weeks of treatment, such as diarrhea/nausea, and should be taken into consideration when interpreting this outcome. Moreover, radiologic data and dactylometer were not included in the study endpoints or assessments.

On the other hand, our study provides valuable real-world data for an early PsA patient population with a non-limiting set of characteristics, examined both from the physicians’ and patients’ perspective, while inclusion of patients from 20 study sites reinforces the generalizability of the results across diverse healthcare settings. Additional strengths of the study lie on the inclusion of a relatively large population (167 eligible patients), the long duration of follow-up (52 weeks), and the use of a comprehensive approach, applying multiple indices to assess PsA articular/extra-articular manifestations, as well as PROs to evaluate disease activity and the impact of PsA on the patient’s daily living and quality of life. The indices selected herein (e.g. cDAPSA) are considered more relevant in PsA than other scores or response criteria (e.g. the ACR20) that have been borrowed from rheumatoid arthritis and were used previously [30].

## Conclusions

In conclusion, based on the results of APROACH, apremilast, when initiated early in the patient journey for PsA, yielded rapid and sustained improvements in all aspects of psoriatic disease manifestations (joint, skin, entheseal), disease-specific health status, and generic HRQoL among biologic-naïve patients treated in routine care settings in Greece. Apremilast demonstrated high drug survival and a safety profile consistent with the product’s label with no unexpected safety signals.

## Supplementary Information

Below is the link to the electronic supplementary material.

Supplementary file1 (DOCX 14 KB)

Supplementary file2 Kaplan-Meier estimated time from initiation to permanent discontinuation of treatment with apremilast (PPTX 67 KB)

Supplementary file3 Multivariable logistic regression analysis for the association of factors of interest with the achievement of minor cDAPSA response at 52 weeks post-baseline. BMI, body mass index; cDAPSA, clinical disease activity in psoriatic arthritis; CI, confidence interval; OR, odds ratio; PsA, psoriatic arthritis (PPTX 46 KB)

## Data Availability

The data that support the findings of this study are not publicly available due to their containing information that could compromise the privacy of patients included in the study.

## Abbreviations

ACR: American college of rheumatology
AE: Adverse event
bDMARDs: Biologic disease-modifying antirheumatic drug
BSA: Body surface area
cDAPSA: Clinical disease activity in psoriatic arthritis
CI: Confidence interval
CRP: C-reactive protein
csDMARD: Conventional synthetic disease-modifying antirheumatic drug
DAPSA: Disease activity index for psoriatic arthritis
DMARD: Disease-modifying antirheumatic drug
DSS: Dactylitis Severity Score
EQ-5D-5L: EuroQol 5-Dimensions 5-Levels
HDA: High disease activity
HAQ-DI: Health assessment questionnaire-disability index
HRQoL: Health-related quality of life
IQR: Interquartile range
LDA: Low disease activity
LEI: Leeds Enthesitis Index
MDA: Moderate disease activity
NRI: Non-responder imputation
NRS: Numerical rating scales
NSAIDs: Non-steroidal anti-inflammatory drugs
PhGA: Physician Global Assessment
PROs: Patient-reported outcomes
PsA: Psoriatic arthritis
PsAID12: Psoriatic arthritis impact of disease 12-item
PtGA: Patient global assessment
SD: Standard deviation
SJC: Swollen joint count
TJC: Tender joint count
tsDMARD: Targeted synthetic DMARD
