# Supplementary material for: Apremilast for biologic-naïve, peripheral psoriatic arthritis, including patients with early disease: results from the APROACH observational prospective study
Source: Rheumatol Int. 2023 Mar 1;43(5):889–902. doi: 10.1007/s00296-022-05269-z (PMC10073163; doi:10.1007/s00296-022-05269-z)
Supplement: Supplementary file 1 — Supplementary file1 (DOCX 14 KB) [file 296_2022_5269_MOESM1_ESM.docx]

**Supplementary Table 1.** Adverse events leading to permanent apremilast discontinuation

| **Adverse events leading to permanent apremilast discontinuation (N=167)** | **n_event_** | **n_pt_ (%)** |
| --- | --- | --- |
| **Description of events by MedDRA v23.1 preferred term** | | |
| Headache | 3 | 3 (1.8) |
| Nausea | 3 | 3 (1.8) |
| Arthritis | 2 | 2 (1.2) |
| Diarrhoea | 2 | 2 (1.2) |
| Fatigue | 2 | 2 (1.2) |
| Insomnia | 2 | 2 (1.2) |
| Back pain | 1 | 1 (0.6) |
| Decreased appetite | 1 | 1 (0.6) |
| Depression | 1 | 1 (0.6) |
| Major depression | 1 | 1 (0.6) |
| Photosensitivity reaction | 1 | 1 (0.6) |
| Psoriasis | 1 | 1 (0.6) |

All of the aforementioned AEs were related to apremilast except for one event of “arthritis” (unrelated), and “psoriasis” (causal relationship was non-assessable); in addition, all events were non-serious apart from “major depression” and one event of “arthritis”.

MedDRA, medical dictionary for regulatory activities; N, total number of patients; n_event_, number of events; n_pt_, number of patients with event
