# Supplementary figures and images for: Apremilast for biologic-naïve, peripheral psoriatic arthritis, including patients with early disease: results from the APROACH observational prospective study
Source: Rheumatol Int. 2023 Mar 1;43(5):889–902. doi: 10.1007/s00296-022-05269-z (PMC10073163; doi:10.1007/s00296-022-05269-z)

## Slide 1
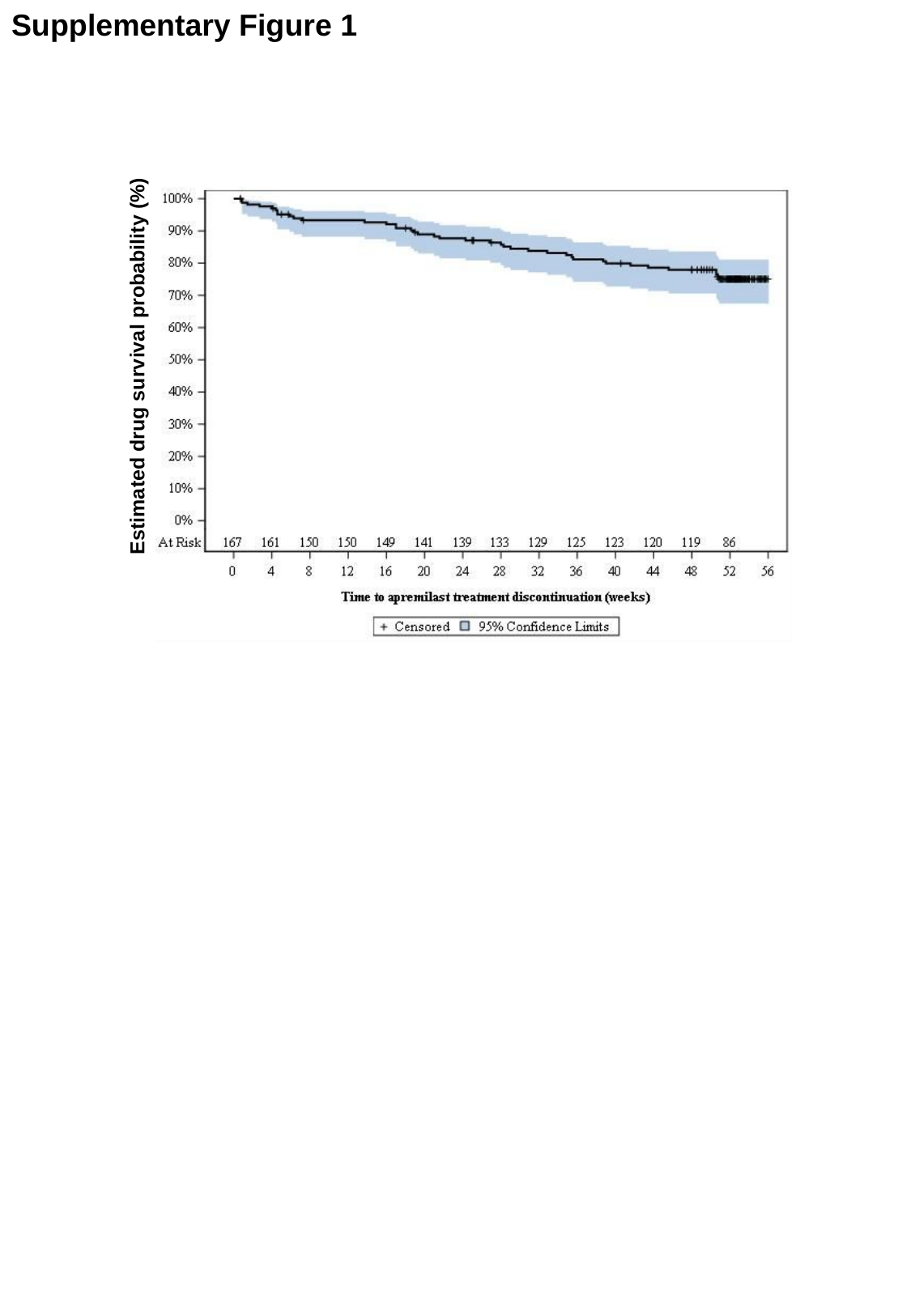

Supplementary Figure 1
Estimated drug survival probability (%)

Supplement: Supplementary file 2 — Supplementary file2 Kaplan-Meier estimated time from initiation to permanent discontinuation of treatment with apremilast (PPTX 67 KB) [file 296_2022_5269_MOESM2_ESM.pptx]
