# Supplementary material for: Apremilast for biologic-naïve, peripheral psoriatic arthritis, including patients with early disease: results from the APROACH observational prospective study
Source: Rheumatol Int. 2023 Mar 1;43(5):889–902. doi: 10.1007/s00296-022-05269-z (PMC10073163; doi:10.1007/s00296-022-05269-z)
Supplement: Supplementary file 3 — Supplementary file3 Multivariable logistic regression analysis for the association of factors of interest with the achievement of minor cDAPSA response at 52 weeks post-baseline. BMI, body mass index; cDAPSA, clinical disease activity in psoriatic arthritis; CI, confidence interval; OR, odds ratio; PsA, psoriatic arthritis (PPTX 46 KB) [file 296_2022_5269_MOESM3_ESM.pptx]

## Slide 1
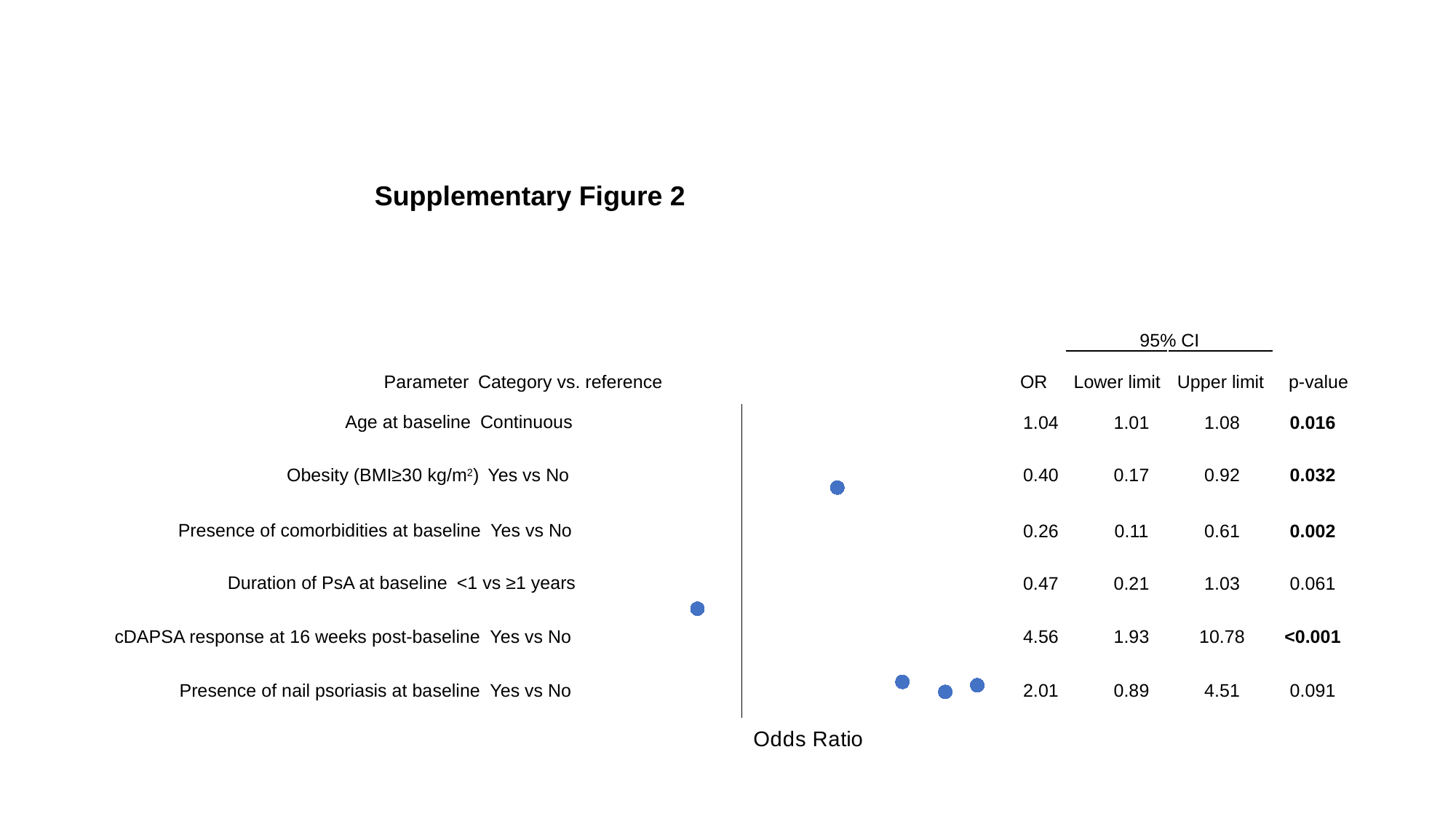

Supplementary Figure 2
| | 95% CI | | |
| --- | --- | --- | --- |
| OR | Lower limit | Upper limit | p-value |
| Parameter | Category vs. reference |
| --- | --- |
### Chart
| Category | | |
|---|---|---|
| Presence of nail psoriasis at baseline | 2.01 | 0.5 |
| cDAPSA response at 16 weeks post-baseline | 4.56 | 1.5 |
| Duration of PsA at baseline | 0.47 | 2.5 |
| Presence of comorbidities at baseline | 0.26 | 3.5 |
| Obesity (BMI≥30 Kg/m2) | 0.4 | 4.5 |
| Age at baseline | 1.04 | 5.5 || Age at baseline | Continuous |
| --- | --- |
| 1.04 | 1.01 | 1.08 | 0.016 |
| --- | --- | --- | --- |
| Obesity (BMI≥30 kg/m2) | Yes vs No |
| --- | --- |
| 0.40 | 0.17 | 0.92 | 0.032 |
| --- | --- | --- | --- |
| Presence of comorbidities at baseline | Yes vs No |
| --- | --- |
| 0.26 | 0.11 | 0.61 | 0.002 |
| --- | --- | --- | --- |
| Duration of PsA at baseline | <1 vs ≥1 years |
| --- | --- |
| 0.47 | 0.21 | 1.03 | 0.061 |
| --- | --- | --- | --- |
| cDAPSA response at 16 weeks post-baseline | Yes vs No |
| --- | --- |
| 4.56 | 1.93 | 10.78 | <0.001 |
| --- | --- | --- | --- |
| Presence of nail psoriasis at baseline | Yes vs No |
| --- | --- |
| 2.01 | 0.89 | 4.51 | 0.091 |
| --- | --- | --- | --- |
